# Climate change and *Aedes albopictus* risks in China: current impact and future projection

**DOI:** 10.1186/s40249-023-01083-2

**Published:** 2023-03-24

**Authors:** Hongmei Liu, Xiaodan Huang, Xiuxia Guo, Peng Cheng, Haifang Wang, Lijuan Liu, Chuanhui Zang, Chongxing Zhang, Xuejun Wang, Guofa Zhou, Maoqing Gong

**Affiliations:** 1 Shandong Institute of Parasitic Diseases, Shandong First Medical University and Shandong Academy of Medical Sciences, Jining, Shandong Province 272033 People’s Republic of China; 2 Program in Public Health, University of California, Irvine, CA 92697 USA; 3 Shandong Center for Disease Control and Prevention, Jinan, 250013 China

**Keywords:** *Aedes albopictus*, Observed climate change, Projected future climate, Observed risks distribution, Projected future risk distribution

## Abstract

**Background:**

Future distribution of dengue risk is usually predicted based on predicted climate changes using general circulation models (GCMs). However, it is difficult to validate the GCM results and assess the uncertainty of the predictions. The observed changes in climate may be very different from the GCM results. We aim to utilize trends in observed climate dynamics to predict future risks of *Aedes albopictus* in China.

**Methods:**

We collected *Ae. albopictus* surveillance data and observed climate records from 80 meteorological stations from 1970 to 2021. We analyzed the trends in climate change in China and made predictions on future climate for the years 2050 and 2080 based on trend analyses. We analyzed the relationship between climatic variables and the prevalence of *Ae. albopictus* in different months/seasons. We built a classification tree model (based on the average of 999 runs of classification and regression tree analyses) to predict the monthly/seasonal *Ae. albopictus* distribution based on the average climate from 1970 to 2000 and assessed the contributions of different climatic variables to the *Ae. albopictus* distribution. Using these models, we projected the future distributions of *Ae. albopictus* for 2050 and 2080.

**Results:**

The study included *Ae. albopictus* surveillance from 259 sites in China found that winter to early spring (November–February) temperatures were strongly correlated with *Ae. albopictus* prevalence (prediction accuracy ranges 93.0–98.8%)—the higher the temperature the higher the prevalence, while precipitation in summer (June–September) was important predictor for *Ae. albopictus* prevalence. The machine learning tree models predicted the current prevalence of *Ae. albopictus* with high levels of agreement (accuracy > 90% and Kappa agreement > 80% for all 12 months). Overall, winter temperature contributed the most to *Ae. albopictus* distribution, followed by summer precipitation. An increase in temperature was observed from 1970 to 2021 in most places in China, and annual change rates varied substantially from -0.22 ºC/year to 0.58 ºC/year among sites, with the largest increase in temperature occurring from February to April (an annual increase of 1.4–4.7 ºC in monthly mean, 0.6–4.0 ºC in monthly minimum, and 1.3–4.3 ºC in monthly maximum temperature) and the smallest in November and December. Temperature increases were lower in the tropics/subtropics (1.5–2.3 ºC from February–April) compared to the high-latitude areas (2.6–4.6 ºC from February–April). The projected temperatures in 2050 and 2080 by this study were approximately 1–1.5 °C higher than those projected by GCMs. The estimated current *Ae. albopictus* risk distribution had a northern boundary of north-central China and the southern edge of northeastern China, with a risk period of June–September. The projected future *Ae. albopictus* risks in 2050 and 2080 cover nearly all of China, with an expanded risk period of April–October. The current at-risk population was estimated to be 960 million and the future at-risk population was projected to be 1.2 billion.

**Conclusions:**

The magnitude of climate change in China is likely to surpass GCM predictions. Future dengue risks will expand to cover nearly all of China if current climate trends continue.

**Supplementary Information:**

The online version contains supplementary material available at 10.1186/s40249-023-01083-2.

## Background

Dengue is a viral infection transmitted to humans through the bite of infected female *Aedes* mosquitoes [1]. Dengue is becoming an increasing global public health threat, not only because no vaccine or effective treatment exists for the disease, but also because of its unpredictable epidemics and its dramatic geographic expansion worldwide due to the aggressive global invasion of the vector *Aedes albopictus* [2–6]. The WHO reported 5.2 million dengue cases in 2019, the largest number ever reported globally, compared to about 0.5 million in 2000 and 2.4 million in 2010. Asia represents approximately 70% of the global burden of the disease [1]. The estimated at-risk population was 3.9 billion in 2010, and risk exists in 129 countries [7–9].

In China, the first dengue outbreak, which was also the first report of dengue, since World War II occurred in 1978 in the southern city of Foshan, Guangdong Province [10]. Before 2000, dengue outbreaks in China were concentrated in a small tropical area in the southern coastal region [11–14]. Prior to 2010, dengue outbreaks moved slowly northward along the southeastern coast [15, 16]; since 2010, however, outbreaks have soared, and in 2013, the wavefront moved to central China [12, 13, 17, 18]. The largest number of outbreaks, in terms of outbreak areas covered, occurred in 2019 and spanned 15 provinces, including Shandong Province in northern China [19–21]. Early dengue outbreaks were likely initiated by internationally imported infections [11, 20]; however, molecular analyses and index case tracking indicate that most recent outbreaks in central and northern China have been caused by domestic travelers returning from dengue-endemic regions of southern China [18, 22, 23]. Although *Aedes aegypti* is believed to be the primary dengue virus vector globally and was responsible for most of the dengue outbreaks in southern China before 2000 [24–27], recent dengue outbreaks in China have been caused almost exclusively by *Ae. albopictus* [28, 29]. In fact, in recent years *Ae. aegypti* has been found only in a few small spots in southern and southwestern China [30, 31], whereas *Ae. albopictus* is found all over China, including in all mild temperate regions in northern China [32, 33].

At the same time, there is a strong link between dengue outbreaks and climatic variability [34–38], since the development and survival of *Aedes* mosquitoes and virus replication depend on environmental, especially climatic, conditions [39–42]. Many studies have modeled the impact of climate change on the future potential regional and global expansion and distribution of dengue virus transmission risk [40, 41, 43, 44]. Nonetheless, dengue outbreaks have expanded into temperate northern China. Global climate change is real, as observed in the past 50 years, and climate change may accelerate the northward expansion of dengue outbreaks in China. However, nearly all dengue risk assessment modeling has used future climate projections from climate models, also known as the General Circulation Models (GCMs)[40, 41, 43–46]. There are different GCMs based on different assumptions, and they produce quite different results [47]. In addition, there are different emissions scenarios [47]. However, uncertainty due to GCMs is rarely assessed, and predicted results may not be validated. A recent study showed that from 1979 to 2021, the Arctic warmed nearly four times faster than the climate model predicted, and the magnitude of temperature increase depended on latitude, indicating that GCMs may severely underestimate global warming [48]. Since we have many years of observational data from meteorological stations across the globe, it would be interesting if not preferable to use real data to make future climate predictions and dengue risk assessments because the results can be tested or validated using currently available observed data [49]. Using observed climatic records has additional advantages. For example, climate changes vary in different regions [48, 49]; temperature increases in the tropics may have a limited impact on dengue risks in these areas, whereas temperature increases in high-latitude temperate zones may push the vector distribution boundary farther north [33, 40, 41, 49], resulting in a major impact on the expansion of the *Aedes* distribution and dengue risk. Using observed climatic data can lead to more accurate estimates of the spatial distribution of climate trends. However, few if any studies have attempted to use real climatic records to assess future dengue risks caused by global climate change [49].

In this study, we analyzed the relationships between the prevalence of *Aedes* mosquitoes and climatic variables in China. We examined the climate trends from 1970 to 2021 based on meteorological observations across China and mapped the spatial variation of these climate trends. Based on these trend analyses, we predicted the potential future climate conditions and *Ae. albopictus* risks in China. Dengue risk seasonality and at-risk populations were also estimated. This study provides an alternative look at the impact of climate change on dengue risk from a different angle.

## Materials and methods

### *Aedes* mosquito data collection

**Fig. 1:**
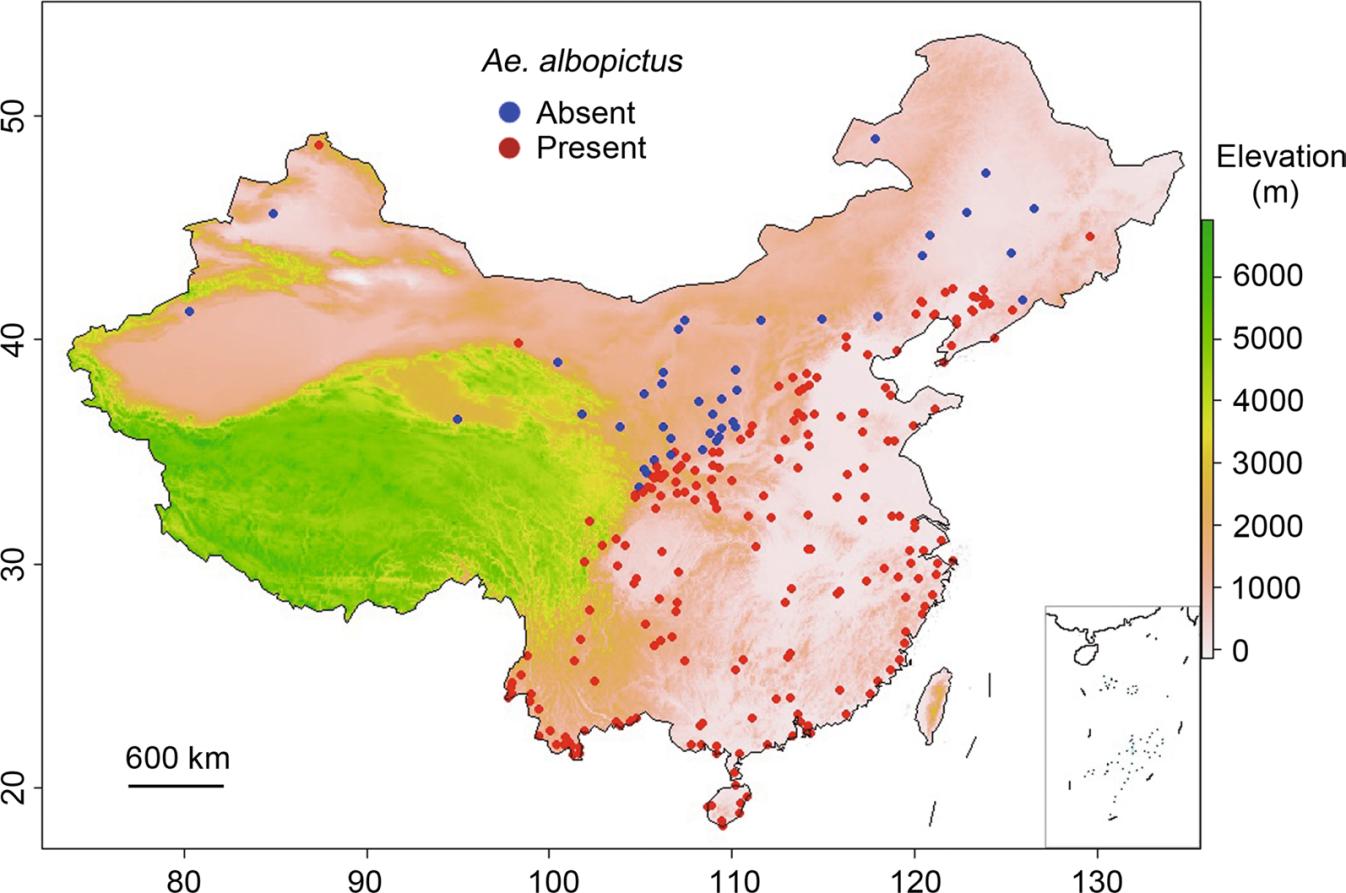
Distribution of *Aedes albopictus* surveillance sites in China

We employed the *Aedes* mosquito collection database established in our previous study [33]. The prevalence of *Aedes* at a site was defined as a place where *Aedes* mosquitoes have been detected. We updated the database by reviewing some recently published work [28, 29, 50–52]. The updated *Aedes* mosquito records included data up to 2021 and covered all provinces/autonomous regions/municipalities in China except Taiwan Province (Fig. 1). The new China CDC *Aedes* surveillance system covers 23 provinces/autonomous regions/municipalities, plus published data from surveillances conducted by provincial CDCs [29, 32, 54]. Most of the surveillances is performed in areas where *Aedes* data are already available, especially in southern China. However, there are several newly *Aedes*-invaded places, especially at high altitude of western Sichuan Province (*Ae. albopictus*) and central Qinghai Province (*Aedes caspius* and *Aedes flavidorsalis*) (Fig. 1). Surveillance sites have also been updated in Tibet Autonomous Region, however, no *Aedes* has been detected in the area (Fig. 1). In this study, we used data for *Ae. albopictus* only; other *Aedes* mosquitoes were not included [54–56]..

Many places in southern China have more than one *Ae. albopictus* records, to avoid redundancy, much of the sampling data from southern China was not included, since *Ae. albopictus* exists everywhere there (Fig. 1). Most of the surveillance results from northern China, especially from areas along the *Ae. albopictus* distribution boundary, were included in the modeling process so that the risk models could identify the environmental/climatic variables that could differentiate mosquito presence/absence sites. A few *Ae. albopictus* present localities are on the eastern slope of the Qinghai-Tibet Plateau, where the elevation is > 3000 m above sea level (Fig. 1). Although other mosquitoes such as *Culex*, *Anopheles*, and other *Aedes* mosquito species such as *Ae. caspius* and *Ae. flavidorsalis* have been found in the central area of the Qinghai-Tibet Plateau, *Ae. albopictus* has not been detected there (Fig. 1). The study included *Ae. albopictus* data from 259 sites for analyses (Fig. 1).

### Environmental and climatic suitability modeling

We updated the machine learning classification and regression tree (CART) model based on the updated *Aedes* database and WorldClim 2.0 data [33, 57]. Details of the climatic and environmental data have been described in our previous study [33]. In brief, the 1970–2000 average monthly climate data for the minimum, mean, and maximum temperature and precipitation were downloaded from WorldClim version 2.1 (https://www.worldclim.org/data/worldclim21.html). The environmental regions were divided into four categories: humid, sub-humid, semiarid, and arid [33]. Climatic zones comprised nine categories: south subtropical, mid-subtropical, north subtropical, warm temperate, mild temperate, cool temperate, plateau subtropical, plateau temperate, and plateau subfrigid [33].

We conducted univariate analyses to examine the relationship between *Ae. albopictus* prevalence and climatic variables using Chi-square automatic interaction detection (CHAID) [58, 59]. The aim was to examine how changes in climatic variables affected the presence/absence of *Ae. albopictus*. CHAID is similar to logistic analysis, but CHAID produces the critical cutoff of predictors and allows for a nonlinear combination of predictors [60]. In addition to overall prediction accuracy, we measured sensitivity and specificity to examine the prediction skewness (bias for presence/absence) and used Yale’s coefficient to measure the association between the observed and predicted prevalence of *Ae. albopictus* by each climatic variable [61, 62]. We did not examine the impact of summer temperatures (June–September) on *Ae. albopictus* prevalence, and we only examined the climatic effect for the same month and the following four months.

For the multivariate analysis, the detailed multi-step modeling process has been described in our previous study [33]. Briefly, after data pre-processing, CART models were developed using a tenfold cross-validation method to predict the potential seasonal (or monthly) distribution ranges of *Ae. albopictus* in China at a high resolution based on environmental-climatic conditions (refer to Additional file 6: Supplement A for modeling details). Since *Ae. albopictus* was found only in northern China from June to September, these months were aggregated as one season for risk analyses. Environmental-climatic suitability for *Ae. albopictus* was predicted as the average predicted suitability probability of the 10 models developed during the tenfold cross-validation modeling process, and the spatial resolution was 30 arcsec or approximately 1 km.

Model performance was measured using prediction accuracy, sensitivity (presence predicted as presence), specificity (absence predicted as absence), and Cohen’s Kappa coefficient [63]. Kappa measures the reliability of agreement between observed and predicted qualitative data and considers the possibility of the agreement occurring by chance.

### Trends in climate change in China, 1970–2021

To examine the heterogeneity of climate trends in China, we collected daily meteorological records from 1970 to 2021 from 90 meteorological stations (Additional file 1: Fig. S1). Since *Aedes* mosquitoes exist nearly everywhere in southern China except the Qinghai-Tibet Plateau, we selected only a representative subset of stations for climate trend analysis. We selected as many stations as possible from northern China, especially near the current margin of the *Aedes* distribution [33], but excluded some stations to avoid oversampling; i.e., if two stations were located very close to each other (< 200 km) we selected only one of the two stations. Daily records were summarized as monthly maximum/minimum/mean temperatures and monthly cumulative precipitation. Trends in monthly data at each station were analyzed using linear regression analysis. Climate trends were measured as the annual change rates in monthly maximum/minimum/mean temperature and annual cumulative precipitation. Due to the large variation in monthly precipitation in different years, trends in monthly precipitation were not analyzed. Climate change trends in China were analyzed by month (temperature) or annually (precipitation) and aggregated based on latitude.

### Climate change and its impact on *Aedes* distribution

To predict future climate distribution, we needed to create a climate trend map of China. Based on our climate trend analyses, we produced the trend distribution map using the geostatistical spatial interpolation method of universal kriging (refer to Additional file 6: Supplement B for modeling details) [64], which assumes a third-order polynomial trend model, i.e., trends in climate change may be linearly or nonlinearly correlated with latitude/longitude. Using this climate trend map and the 1970–2000 mean climate as the baseline, we predicted the temperature and precipitation distributions in China for 2020, 2050 and 2080, a typical risk projection framework [40, 41]. We compared the projected temperature increases in 2050 and 2080 between this study and the GCMs using 2000 as the baseline [41].

We used the suitability models established earlier to predict the *Aedes* distribution in each month based on the 2020, 2050 and 2080 climatic projections. *Ae. albopictus* risk was measured as the probability of presence of *Ae. albopictus*.

We estimated the at-risk population for different projections based on 2010 census data for each county in China and the 2020 total population [65]. Since there was no updated population distribution for 2020, we projected the 2020 population distribution to be the same as that in 2010. Because projecting the 2050 and 2080 total populations and population distributions would create additional uncertainty, i.e., we do not know the future population movement and growth across China, we used the 2020 total population as the base population and fixed it for 2050 and 2080. We were aware of the potential bias for estimating the future at-risk population based on the 2020 population, but this was the best method upon which we could rely. If the total population decreases by 2050 and 2080, the at-risk population will need to be adjusted accordingly.

All data analyses were conducted using R 4.2.1 (R Foundation for Statistical Computing, Vienna, Austria) except universal kriging, which was performed using ArcGIS Pro 3.0.0 (ESRI, Redlands, CA, USA). The following R packages were used in this study: for raster image reading and risk mapping, we used the raster and crop methods within the *rasterImage* and *sp* packages; and for regression tree modeling, we used the ctree and rpart methods within the *rpart*, *party*, and *caret* packages.

## Results

### The impact of climatic variables on *Ae. albopictus* prevalence in China

Univariate analyses revealed that wintertime (November–February) mean and maximum temperature were strongly correlated with *Ae. albopictus* presence in the following three months (Yale’s correlation coefficient ranged from 0.84 to 1.00). October mean and maximum temperature were also important predictors of *Ae. albopictus* presence (Yale’s *R* 0.56–0.94) (Table 1); the results indicated that the higher the temperature was, the higher the prevalence (Additional file 2: Fig. S2). The minimum temperature was moderately associated with *Ae. albopictus* prevalence (Yale’s *R* 0.15–0.95) (Table 1; Additional file 2: Fig. S2). Precipitation was moderately correlated with *Ae. albopictus* prevalence during April–September (Yale’s *R* 0.15–0.91) (Table 1; Additional file 2: Fig. S2).

**Table 1:** CHAID univariate analysis of the correlation between climatic variable and *Aedes albopictus* prevalence

Climatic variable	Month	*Ae. albopictus* prevalence by month								
		Jan	Feb	Mar	Apr	May	Jun–Sep	Oct	Nov	Dec
Mean temperature	Jan	1	0.99	0.94	0.88	0.77				
	Feb		0.94	0.91	0.85	0.76				
	Mar			0.88	0.84	0.77				
	Apr				0.7	0.72				
	Oct	0.91	0.9					0.63	0.78	0.94
	Nov	1	0.92	0.84					0.9	0.92
	Dec	0.95	0.97	0.92	0.88					0.97
Maximum temperature	Jan	0.91	0.89	0.91	0.85	0.79				
	Feb		0.85	0.93	0.8	0.74				
	Mar			0.88	0.68	0.72				
	Apr				0.59	0.65				
	Oct	0.92	0.93					0.56	0.7	0.94
	Nov	0.94	1	0.93					0.88	0.92
	Dec	0.94	0.95	0.97	0.92					0.97
Minimum temperature	Jan	0.87	0.22	0.15	0.29	0.47				
	Feb		0.95	0.85	0.88	0.75				
	Mar			0.79	0.87	0.75				
	Apr				0.85	0.83				
	Oct	0.82	0.95					0.57	0.84	0.94
	Nov	0.94	0.88	0.85					0.88	0.92
	Dec	0.84	0.95	0.84	0.95					0.97
Precipitation	Jan	0.16	0.36	0.34	0.79	0.62				
	Feb		0.3	0.39	0.91	0.67	0.64			
	Mar			0.33	0.7	0.5	0.15			
	Apr				0.54	0.76	0.83			
	May					0.7	0.57			
	Jun–Sep	-0.05					0.55	-0.03	0.15	0.16
	Oct	0.74	0.54					0.44	0.58	0.48
	Nov	0.49	0.26	0.2					0.56	0.3
	Dec	0.2	0.33	0.33	0.87					0.32

Numbers represent Yale’s correlation coefficient between *Ae. albopictus* prevalence and climatic variables by prevalence at different months against climatic variables at different months. Empty cell means not examined because we assume that climatic effects lag up to four months. Since *Ae. albopictus* has been detected from June to September everywhere where it was found, therefore, May to September temperature is assumed to be perfect for *Ae. albopictus*, thus they were not included in the analyses

### *Aedes albopictus* distribution modeling

**Fig. 2:**
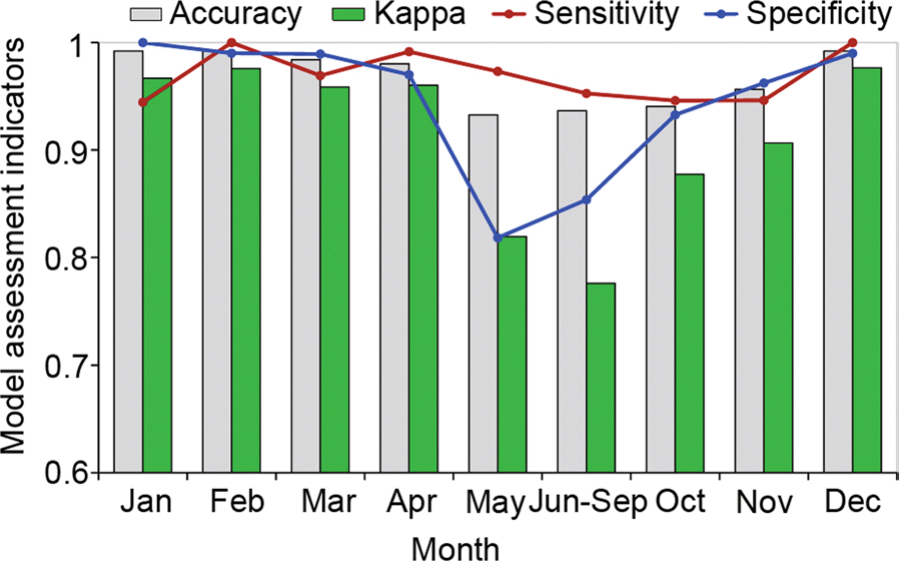
Agreement between model predicted and observed *Aedes albopictus* presence/absence

Multivariable modeling with tenfold cross-validation indicated that the models predicted the existing *Ae. albopictus* sites with very high accuracy (Fig. 2). The accuracy of the predictions ranged from 93.2% in May to 99.2% in February. Kappa agreement between observed and predicted *Aedes* prevalence ranged from 0.78 for June–September to 0.98 in February indicating almost perfect agreement (> 0.80 considered perfect). Sensitivity ranged from 94.6% (November) to 100% (February), and specificity ranged from 81.8% (May) to 100% (January) (Fig. 2).

Variable importance analyses found that winter to early spring (October–February) temperature were the most important factors that determined the presence of *Ae. albopictus*, and the relative influences of each variable on *Ae. albopictus* presence were very similar from December to March (ranging from 15 to 20%) (Table 2). A greater number of variables influenced the *Ae. albopictus* distribution from April to November and environmental/climatic zones were only important for determining June–October *Ae. albopictus* distribution (Table 2). The overall total influence of climatic variable in each month varied from no influence to 82% (Table 3). October to February temperatures contributed the most (range 19–82%, mean ± standard deviation (*SD*): 44.3 ± 20.5%), followed by summertime (June–September) precipitation (range 10–15%, mean 12.8 ± 2.2%) (Table 3).

**Table 2:** Relative influence (RI) of climatic/environmental variables to the prediction of *Aedes albopictus* presence in different months

Presence in January		Presence in February		Presence in March		Presence in April		Presence in May	
Variables	RI	Variables	RI	Variables	RI	Variables	RI	Variables	RI
Mean temp 10	0.20	Min temp 1	0.17	Max temp 1	0.17	Min temp 2	0.16	Min temp 4	0.17
Mean temp 1	0.17	Min temp 2	0.17	Max temp 2	0.17	Mean temp 12	0.16	Min temp 5	0.16
Mean temp 11	0.17	Max temp 11	0.17	Max temp 11	0.16	Min temp 1	0.15	Min temp 3	0.15
Mean temp 12	0.16	Mean temp 1	0.17	Max temp 12	0.18	Min temp 12	0.15	Mean temp 1	0.15
Max temp 1	0.15	Mean temp 2	0.16	Mean temp 1	0.16	Max temp 12	0.15	Mean temp 2	0.15
Max temp 11	0.15	Mean temp 12	0.16	Mean temp 12	0.16	Mean temp 1	0.15	Mean temp 4	0.15
						Precip 1	0.02	Max temp 2	0.02
						Precip 2	0.02	Max temp 3	0.02
						Precip 3	0.02	Max temp 1	0.01
						Precip 4	0.01	Mean temp 3	0.01
						Precip 12	0.01	Precip 3	0.01

Presence from June to September					Presence in October		Presence in November		Presence in December	
Variables	RI		Variables	RI	Variables	RI	Variables	RI	Variables	RI
Environmental zone	0.21		Mean temp 8	0.02	Mean temp 10	0.17	Mean temp 10	0.17	Min temp 12	0.17
Precip 6	0.12		Max temp 6	0.01	Min temp 10	0.16	Min temp 10	0.16	Max temp 11	0.17
Precip 4	0.1		Max temp 7	0.01	Min temp 9	0.15	Min temp 9	0.15	Mean temp 11	0.17
Precip 5	0.1		Max temp 8	0.01	Max temp 10	0.15	Mean temp 9	0.15	Mean temp 12	0.17
Precip 7	0.1		Precip 3	0.01	Mean temp 9	0.15	Min temp 5	0.14	Max temp 12	0.16
Climatic zone	0.09		Precip 8	0.01	Min temp 5	0.14	Max temp 10	0.14	Mean temp 10	0.16
Min temp 6	0.04				Precip 4	0.02	Precip 4	0.02		
Mean temp 6	0.04				Min temp 8	0.01	Precip 10	0.02		
Min temp 5	0.03				Max temp 9	0.01	Min temp 8	0.01		
Min temp 8	0.03				Precip 5	0.01	Max temp 9	0.01		
Mean temp 5	0.03				Precip 6	0.01	Precip 5	0.01		
Mean temp 4	0.02				Precip 9	0.01	Precip 6	0.01		
Mean temp 7	0.02				Climatic zone	0.01	Precip 9	0.01		

Variable names – “Max temp” represents maximum temperature; “Min temp” represents minimum temperature; and “Mean temp” represents mean temperature. Numbers following variable names represent months, i.e., 1–12 represent January to December. Total relative influence (RI) for each month is 1

**Table 3:** The overall relative influence (RI) of climatic/environmental variables in each month to the presence of *Aedes albopictus*

Month	Minimum temperature	Mean temperature		Maximum temperature	Precipitation
January	**0.32**	**0.8**		**0.34**	0.02
February	**0.33**	**0.31**		0.19	0.02
March	0.15	0.01		0.02	0.04
April	0.17	0.17			0.15
May	**0.47**	0.03			0.12
June	0.04	0.04		0.01	0.14
July		0.02		0.01	0.1
August	0.05	0.02		0.01	0.01
September	**0.3**	**0.3**		0.02	0.02
October	**0.32**	**0.66**		**0.29**	0.02
November		**0.35**		**0.66**	
December	**0.32**	**0.82**		**0.49**	0.01
Environment and climate zone		RI			
Climatic zone		0.1			
Environmental zone		**0.21**			

The total RI was 9.0 representing the nine study months. Empty cell means insignificant and numbers with bold font represent contributions > 20%

### Climate changes from 1970 to 2021

**Fig. 3:**
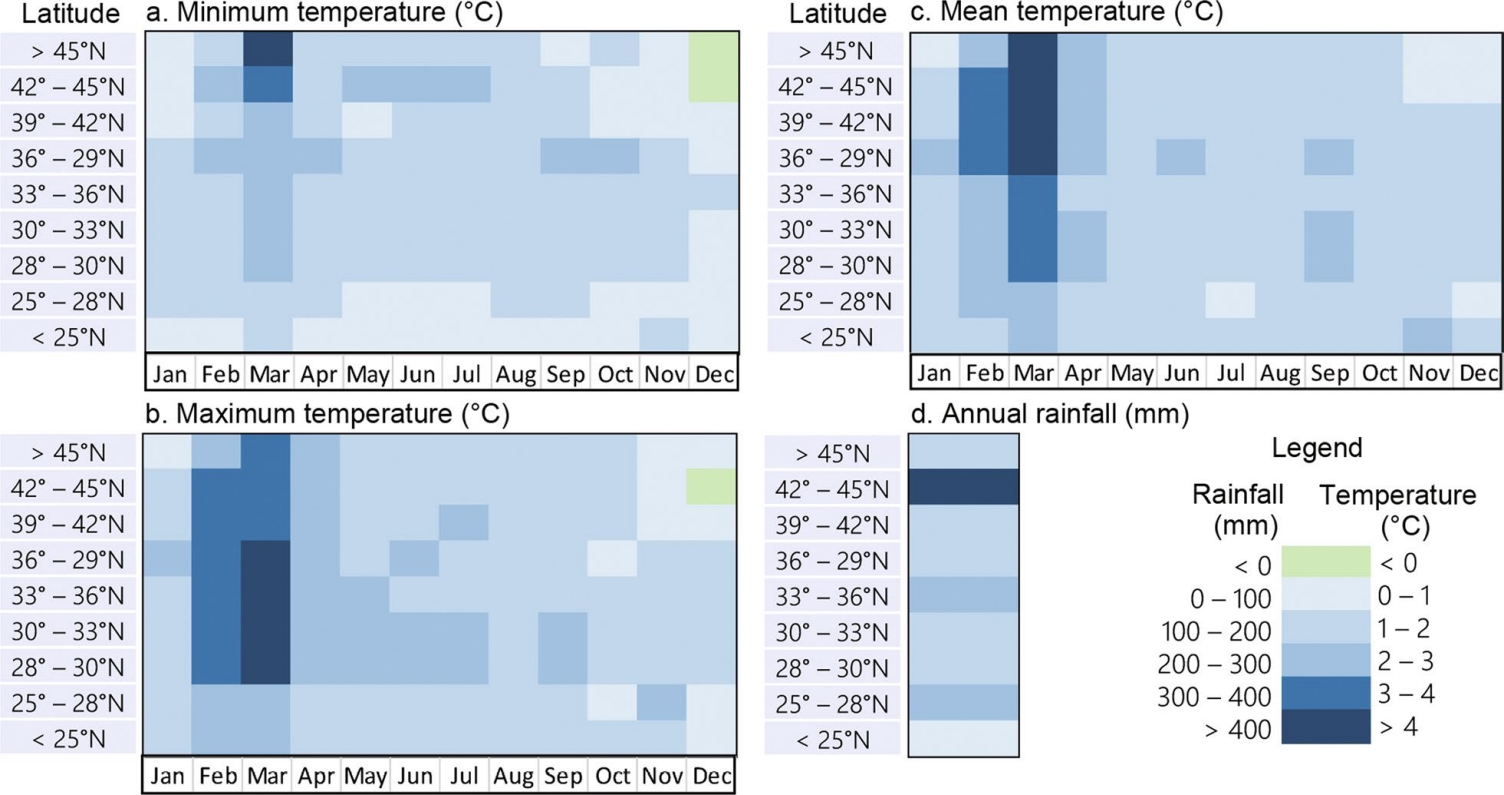
Changes in monthly temperature and annual precipitation by latitude

**Fig. 4:**
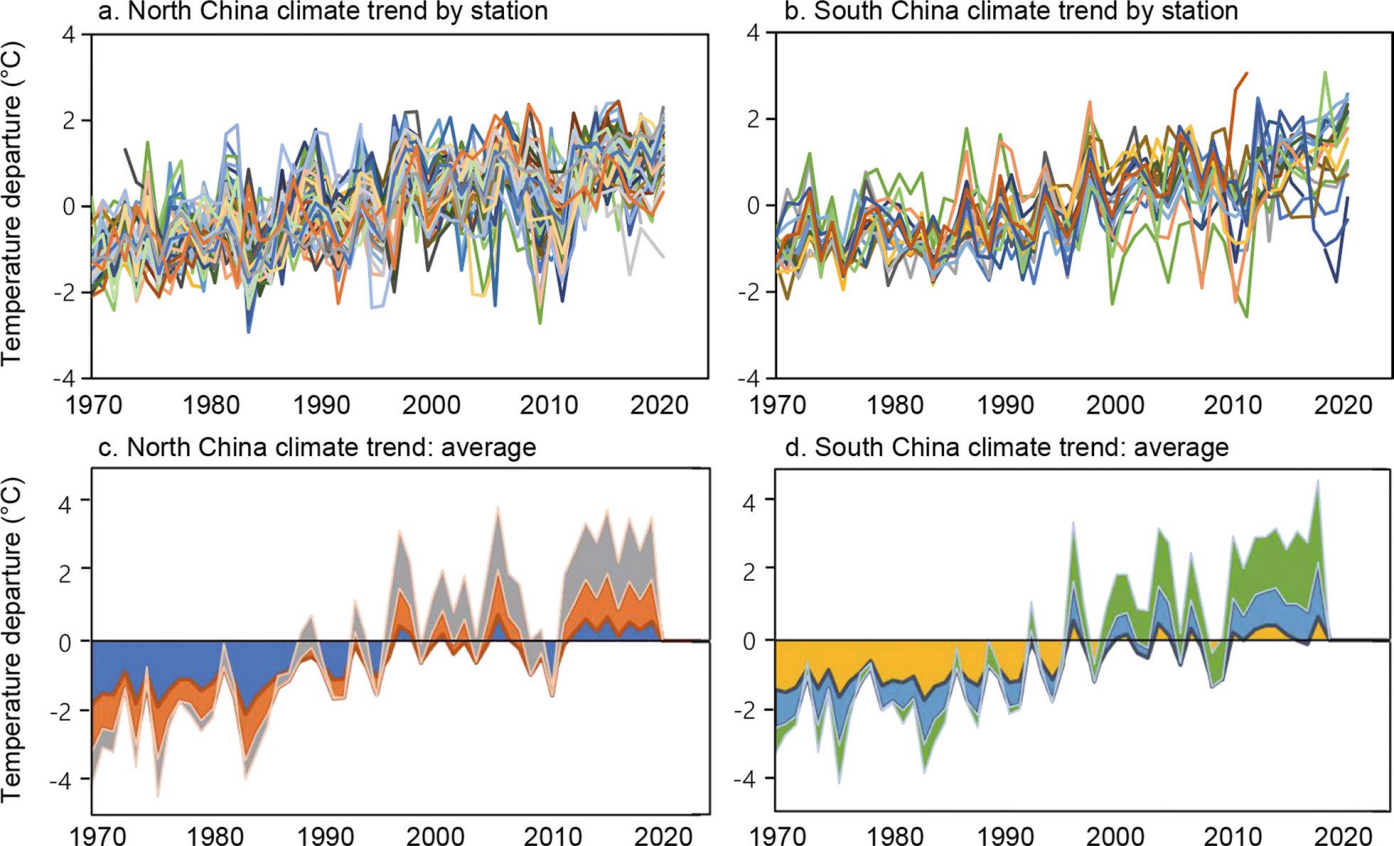
Monthly mean temperature anomaly in different places (top panel) and overall mean (± standard deviation, bottom panel) in central and northern China (left panel) and in southern China (right panel)

We analyzed changes in monthly temperature and annual precipitation for the study period. We found that in general, temperature increases were more pronounced in central and northern China than in the southern subtropical areas and were greater in spring (February–April) than in winter (October–December) (Fig. 3). The minimum temperature increased the most, approximately 3–4 °C, in high-latitude areas in the north in March, while the maximum temperature increased by 3–4 °C across central China from February to March. The greatest increase in the mean temperature (4.7 °C in March) was similar to that of the maximum temperature (4.3 °C in March) but was more pronounced in the north (Fig. 3). For example, the average increase in the maximum temperature in March was 3.5 °C in the north > 39°N and 4.1 °C in the south-central 28–39°N, whereas mean temperature in March increased 4.5 °C in the north > 36°N and 3.5 °C in the south-central 28–36°N (Fig. 3). The winter temperature increase was minimal (Fig. 3). In most places and months, the temperature increased by approximately 1–2 °C (Fig. 3); however, inter-station variation was large (Additional file 3: Fig. S3). For example, in Ejin Qi of Inner Mongolia Autonomous Region, north of the current *Ae. albopictus* northern boundary, the minimum temperature increased at least 3 °C in almost all months and more than 5 °C in two months (Additional file 3: Fig. S3a), while in Datong in Shanxi Province, which lies just on the *Ae. albopictus* distribution northern boundary line, the minimum temperature decreased by approximately 2 °C in several months (Additional file 3: Fig. S3a). Overall, the normalized (anomaly) mean temperature showed very similar trends in central, western and northern China, i.e., north of (including) Xuzhou (Jiangsu Province), Zhengzhou (Henan Province), and Xi’an (Shannxi Province) cities, plus Gansu, Xinjiang and Tibet Province/Autonomous Regions (Fig. 4a), while a few stations in southern China showed almost no change in the mean temperature from 1970 to 2021 (Fig. 4b). The major changes in the mean temperature started in 1990, i.e., the mean temperature was below normal before 1990 and above normal after 1990 (Fig. 4c, d). The trends were very similar for southern (mean increase of 0.0432 °C/year, R^2^ = 0.72) and northern (mean increase of 0.0436 °C/year, R^2^ = 0.71) China (Fig. 4c, d).

Monthly precipitation varied greatly, and we did not find consistent trends (results not shown). However, analysis of annual precipitation found that overall precipitation increased > 100 mm during the study period in most regions (Fig. 3d). Similar to temperature, trends in precipitation varied greatly among stations (Additional file 3: Fig. S3b). For example, in Beijing, although annual precipitation increased in the past two decades, the overall change in precipitation from 1970 to 2021 was nearly zero (annual change rate − 0.162 mm); however, in many other places, precipitation increased considerably in the past 10 years (Additional file 3: Fig. S3b).

### Projection of future climatic conditions and distribution of *Ae. albopictus*

Despite variations, the observed annual temperature anomaly showed a clear linear increase from 1970 to 2021 (Temperature = 0.0434, Year–86.146, *R*^*2*^ = 0.71) (Fig. 4). Based on the linear regression model, we predicted that the mean temperature will increase approximately 2.17 °C (range 1.88–2.46 °C) in China by 2050 from the 2000 baseline, compared to the GCM prediction of 0.98–1.36 °C increase globally by 2050 [47], i.e., GCMs underestimated approximately 1 °C increase in temperature. Similarly, we predicted a 3.47 °C (range 3.10–3.93 °C) increase in the mean temperature by 2080 based on meteorological observations, while GCMs predicted 1.06–2.39 °C increase by 2080 [47].

**Fig. 5:**
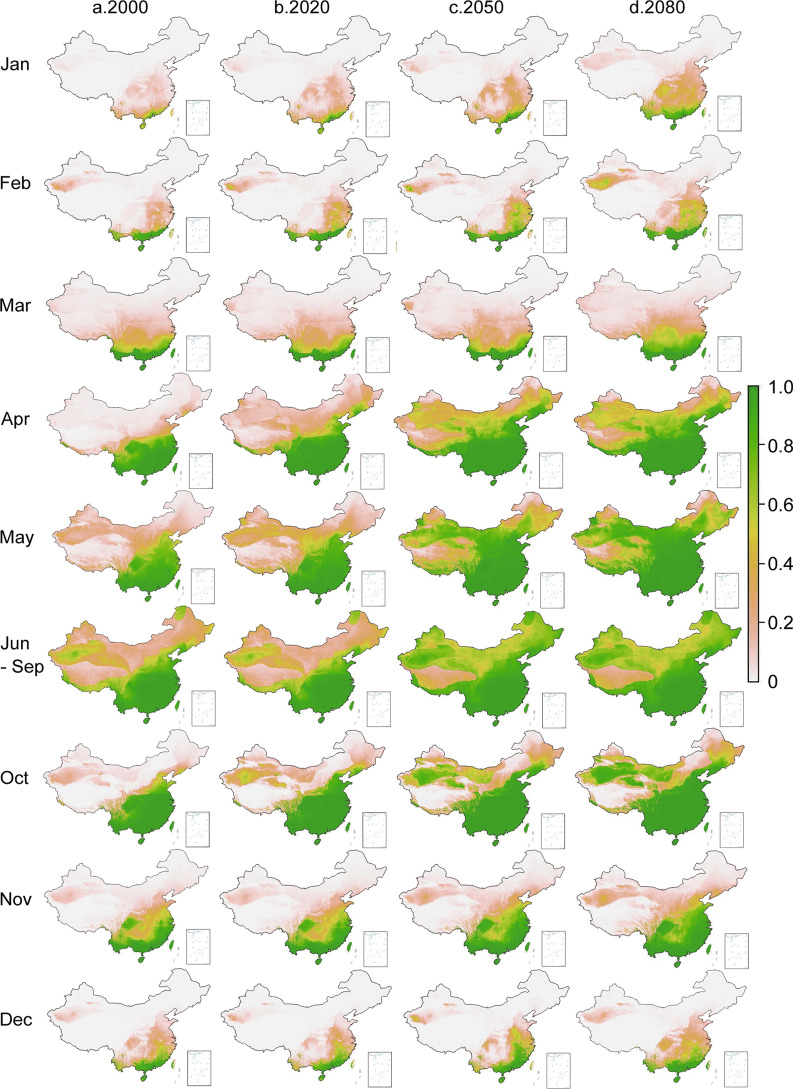
Model estimated baseline distribution of *Aedes albopictus* in 2000 (**a**) and projected probability distribution of *Ae. albopictus* in different months/seasons in 2020 (**b**), 2050 (**c**), and 2080 (**d**)

Figure 4 shows the estimated baseline (2000) distribution of the *Ae. albopictus* in China, the updated current distribution (2020), and the projected distributions for 2050 and 2080 based on predicted climate changes (Additional file 4: Fig. S4, Additional file 5: Fig. S5). The major changes in the *Ae. albopictus* distribution from 2000 to 2020 were the slight expansion in north-central and northeastern China in April and May (Fig. 5a, b). However, major changes in the *Ae. albopictus* distribution were projected for April–November by 2050 and 2080 (Fig. 5c, d). Currently, *Ae. albopictus* distribution is limited to north-central and a small portion of northeastern China; by 2050, if the current trend in climate change continues, *Ae. albopictus* may be found in most parts of northern China, mostly in the summer (June–September) and possibly in April, May, and October (Fig. 5c, d). Expansion in the *Ae. albopictus* distribution in the winter (December–February) was limited, even for 2050 and 2080 (Fig. 5c, d).

After consideration of the conditions for vector development and dengue virus growth (replication), and in consideration of current dengue outbreak areas in China, we estimated a population of approximately 960 million at risk of *Ae. albopictus* in 2020. Based on the 2020 total population in China and the population distribution from the 2010 census, we estimated that the at-risk population will increase to approximately 1.2 billion by 2050, with 1.02 billion at high risk (risk probability > 0.5), which covers south China to the west up to Yunnan and Sichuan provinces, to the north up to southern parts of Hebei, Shanxi and Shannxi provinces, and an additional 180 million at moderate to low risk (risk probability ≤ 0.5), which includes a small part of southern Gansu Province, northern parts of Shanxi and Hebei provinces, Liaoning Province and southeastern Inner Mongolia Autonomous Region (Fig. 5). The risk maps for 2080 are similar to those for 2050 (Fig. 5). We must note that our estimates of the at-risk population were based on current census data (2010 distribution and 2020 total population) and did not include possible future increases/decreases in total population or changes in the population distribution.

## Discussion

Many studies have predicted future distributions of *Aedes* mosquitoes and risks of dengue, including regional- and global-level predictions, based on GCMs of different climate change scenarios [40, 41, 43–46]. Since different models yield quite different results, it is difficult to assess the uncertainty of the predictions and to validate the modeling results. A study of climate change in the Arctic found that the actual increase in temperature from 1979 to 2021 was 4 times of that predicted by climate model [48], indicating the uncertainty of GCM predictions and the importance of observational data. To our knowledge, this is the first study to use actual observed climate trends to predict the future distribution of dengue risks worldwide. We found strong trends (measured by linear regression and correlation coefficient) in climate warming across nearly all meteorological observations in China, which makes our climate change predictions reliable if the current trend holds; the fixed annual change rates are similar to the assumptions for the climate models [47], but our predictions are supported by over 50 years of observations. We found that the temperature increased the most from February to April. We also found strong correlations between the prevalence of *Ae. albopictus* and observed winter to early spring temperature, the months with the greatest temperature increase, indicating that the warming temperature may have major impact on the northward expansion of the *Ae. albopictus* distribution. The projected temperatures in 2050 and 2080 in this study were approximately 1–1.5 °C higher than those projected by GCMs. Our model predicted that *Ae. albopictus* risk will expand to nearly all populated areas in China and the risk season will expand from June–September to April–October by 2050, likely due to the substantial increase in temperature from February to April. We estimated that the current population at risk of *Ae. albopictus* in China is approximately 960 million, or approximately 68% of the total population, and will reach 1.2 billion or approximately85% of the current population by 2050.

Dengue outbreaks in China have been reported in Shandong, Henan, and Chongqing provinces [19–21, 32, 54], which are not far from the current known northern *Ae. albopictus* distribution boundary [33], indicating that the risk of dengue outbreaks can reasonably be predicted by the distribution of the vector mosquito *Ae. albopictus*. Since *Ae. albopictus* is nearly the sole vector for recent dengue outbreaks in China [28, 29], the observed dengue outbreaks in northern China also demonstrate the urgency for updated *Ae. albopictus* surveillance in northern China, which is currently not available [28, 29, 50–52]. The increase in temperature in northern China warrants the need to use observed climate changes to examine the impact of these changes on dengue outbreak risks, both in China and worldwide. Given the wide distribution of imported dengue cases all over China [29, 66], our study is a timely example of such an approach, and our results show the power of using observed meteorological records for predicting future dengue outbreak risks.

Incidentally, it is interesting to note that *Aedes* species other than *Ae. albopictus* have also been observed in China. The known major dengue vector *Ae. aegypti* has been reported in Yunnan in southwestern China bordering Myanmar [53, 67], where dengue outbreaks have been reported. *Ae. albopictus* has also been reported in the same area [53]. Our model predicted very high risk of dengue outbreaks in Yunnan Province nearly year-round in the China-Myanmar border area. *Aedes vexans* has been reported from Heilongjiang Province [56], a northeastern province bordering the Democratic People's Republic of Korea, Russia and Mongolia, far north of the current *Ae. albopictus* northern boundary. In addition, *Ae. caspius* and *Ae. flavidorsalis* have been reported in central Qinghai Province, the core area of the Qinghai-Tibet Plateau [55, 68]. *Culex* and *Anopheles* mosquitoes have also been reported in these places [68]. The vector status of these *Aedes* mosquitoes is unknown, as is the impact of climate change on the distribution and vector status of these *Aedes* mosquitoes. Furthermore, *Culex pipiens pipiens*, *Culex pipiens pallens*, *Culex pipiens quinquefasciatus* and its hybrids have in recent years established populations in Lhasa city, Tibet Autonomous Region, 3700 m above sea level [69]; whether they can transmit diseases is unknown. However, malaria and malaria vectors have been reported in Motuo County, Tibet [70, 71], indicating the possibility of pathogen transmission at high altitudes, and climate change may enhance or support the transmission of pathogens in the highlands.

There are several limitations to this study. Although validation analysis showed that our model had high power to predict the observed presence of *Ae. albopictus*, the suitability model predicted the potential distribution of *Ae. albopictus* in the Taklamakan Desert area of southwestern Xinjiang in northwestern China, which is likely due to the lack of *Ae. albopictus* surveillance data and the sparse distribution of meteorological records in the area; the predicted future climate in this area was likely biased by the use of ground observations from other stations. In this context, mosquito surveillance should be enhanced by setting up more monitoring stations in western and northeastern China (see Fig. 1), and better surveillance coverage may improve the power of model predictions and capture the potentially newly invaded areas by *Ae. albopictus* in China. Some dengue risk models have used human population density as an independent risk predictor [9, 72, 73], which may reduce the uncertainty of model predictions; since the desert is a no-man’s land, the dengue risk will be zero. In this study, we used only climatic and environmental data [33]; therefore, we only predicted the climate suitability for dengue transmission. On the other hand, the environmental variables already included humid, sub-humid, semiarid, and arid regions as an independent variables [33], so the predicted suitability in the desert area is likely due to the lack of *Ae. albopictus* surveillance data from arid areas. In addition, adding the human population as a variable may not have a major impact on the overall results, because population density is high in northeastern China but no *Ae. albopictus* has been detected in the area, likely due to the low temperature. Since future trends in climate change may not be the same as those in the last 50 years, we cannot necessarily assume a fixed trend, i.e., the prediction of future climate change has uncertainty. However, most if not all climate models use the fixed emission assumption over time, although they allow for different emissions scenarios [40, 41, 43]. Since we used observed climatic data, the projected distribution of *Ae. albopictus* can be adjusted or the model calibrated when future data are updated; this may be a viable solution to address the uncertainty of future climate change. Finally, in addition to the uncertainty in future global climate change, future population growth in China is also uncertain especially by 2050 and beyond, recent birth/death records show a substantial downward trend in population growth in China. Since it is difficult to predict both the future population trend in China and population movement (thus population distribution), therefore there is an uncertainty for the prediction of future at-risk populations.

## Conclusions

Dengue outbreaks have intensified in temperate northern China, in addition to the near endemic status of dengue in southern China. Climate change has also intensified in the past 50 years [74]. *Ae. albopictus* is rapidly expanding its distribution [41, 75, 76]. This expanded distribution, fueled by increased temperatures, will likely enhance dengue transmission especially in high-latitude and high-altitude areas, as observed from field vector surveillance. Dengue outbreaks, an old threat, have become a new challenge for future prevention and control efforts in the era of climate change. A climate-based early warning system is urgently needed so that risks can be assessed in a timely manner to support preparedness for future outbreaks.

## Supplementary Information

**Additional file 1: Figure S1**. Distribution of meteorological stations in China where 1970–2021 climatic records were obtained.

**Additional file 2: Figure S2**. Univariate analyses of relationship between *Ae. albopictus* presence and climatic variables. a) Monthly mean temperature (°C); b) Monthly maximum temperature (°C); and c) Monthly minimum temperature (°C). Acc stands for accuracy. Yale represents Yale’s association. Dash line represents the optimal cutoff of temperature.

**Additional file 3: Figure S3**. a) Examples of changes in monthly minimum temperature in different places; b) Examples of changes in annual precipitation in different places.

**Additional file 4: Figure S4**. Distribution of annual changes in mean (a), maximum (b), and minimum (c) temperature from January to December for the period 1970–2021.

**Additional file 5: Figure S5**. Universal Kriging estimated annual changes in precipitation (mm) in China.

**Additional file 6**: A Machine Learning Classification and Regression Trees (CART) for Aedes albopictus distribution modeling. B. Universal Kriging for spatial interpolation of annual climate changes.

## Data Availability

All data generated or analyzed during this study are included in this published article and its additional files.

## Abbreviations

GCMs: General circulation models
CART: The machine learning classification and regression tree
CHAID: Chi-square automatic interaction detection
