# Supplementary figures and images for: Climate change and Aedes albopictus risks in China: current impact and future projection
Source: Infect Dis Poverty. 2023 Mar 24;12:26. doi: 10.1186/s40249-023-01083-2 (PMC10037799; doi:10.1186/s40249-023-01083-2)

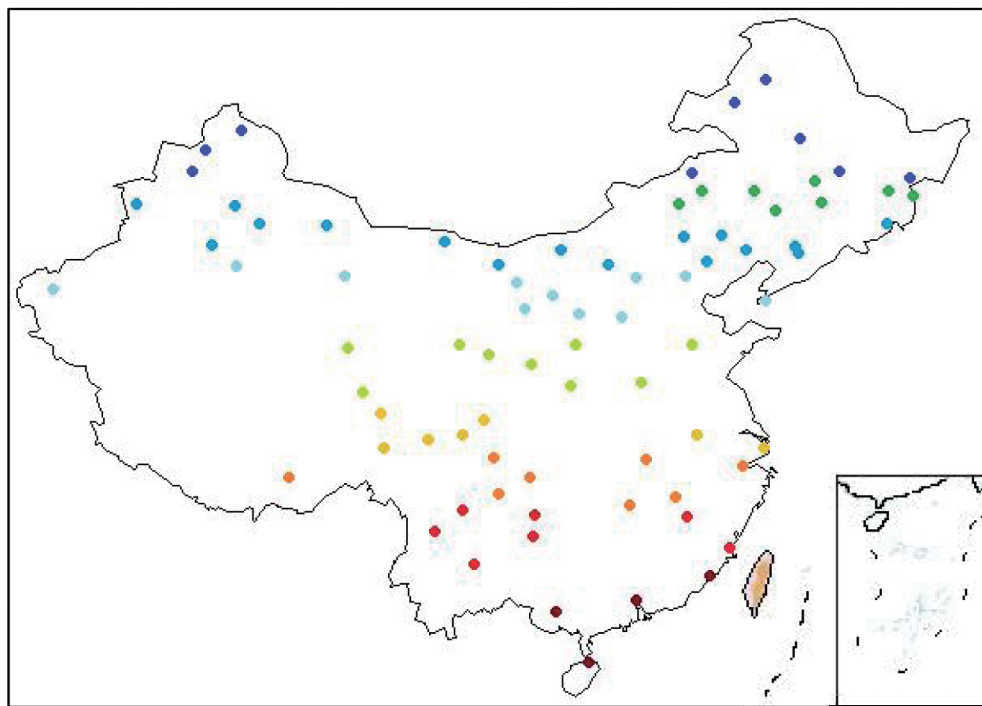

Supplement: Supplementary file 1 — Additional file 1: Figure S1. Distribution of meteorological stations in China where 1970–2021 climatic records were obtained. [file 40249_2023_1083_MOESM1_ESM.pdf]

a. Examples of changes in minimum temperature

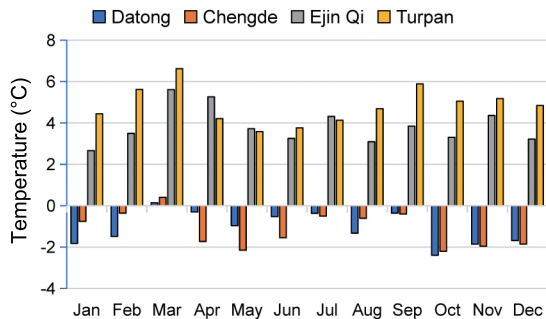

b. Examples of changes in annual precipitation

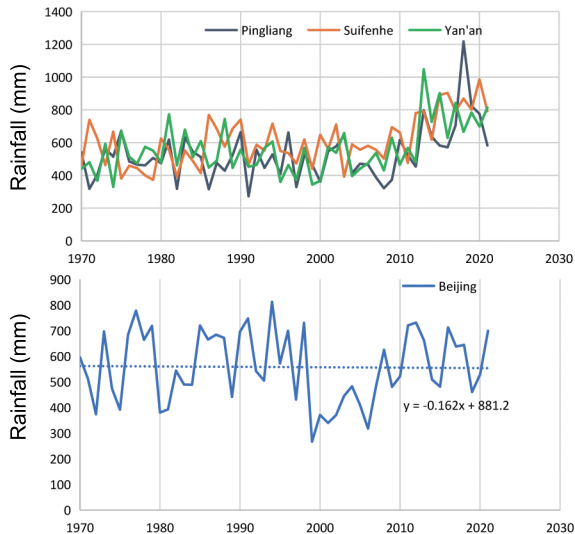

Supplement: Supplementary file 3 — Additional file 3: Figure S3. a) Examples of changes in monthly minimum temperature in different places; b) Examples of changes in annual precipitation in different places. [file 40249_2023_1083_MOESM3_ESM.pdf]

A. Mean temperature B. Maximum temperature C. Minimum temperature

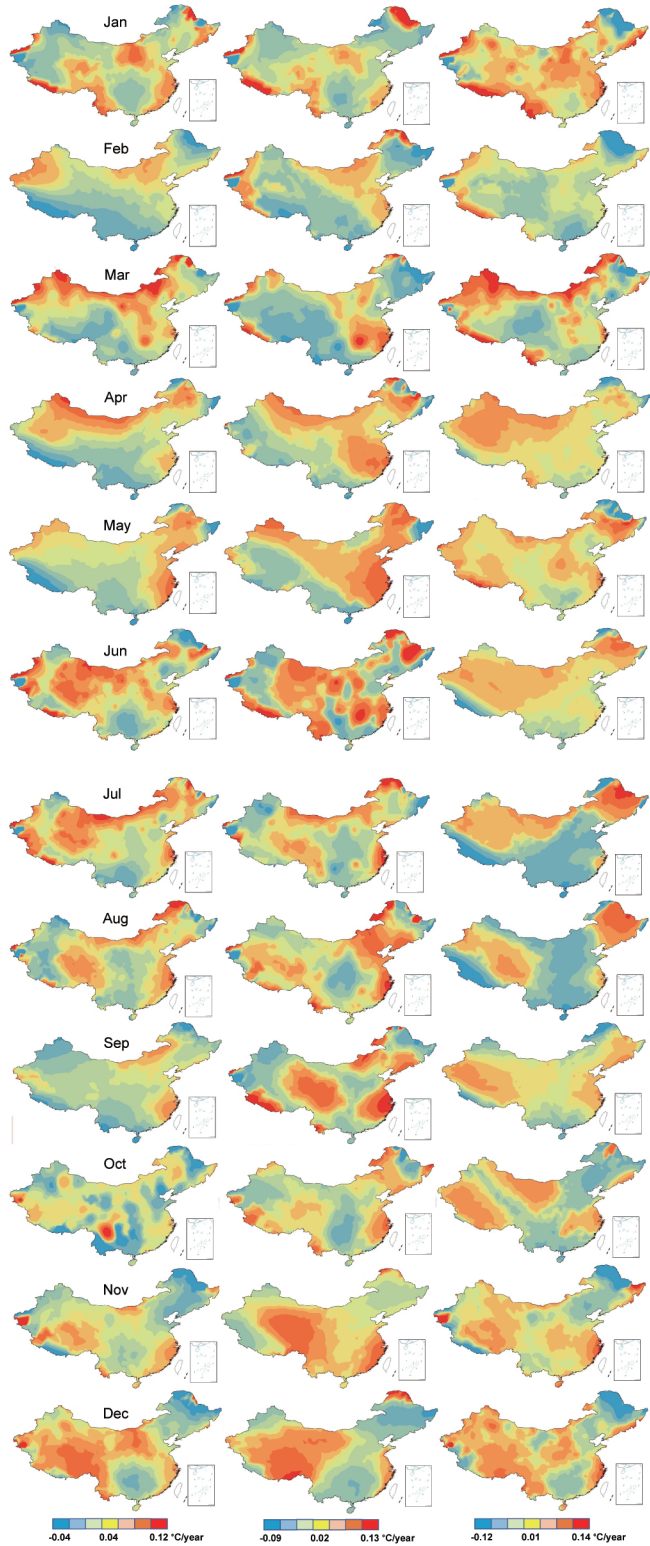

Supplement: Supplementary file 4 — Additional file 4: Figure S4. Distribution of annual changes in mean (a), maximum (b), and minimum (c) temperature from January to December for the period 1970–2021. [file 40249_2023_1083_MOESM4_ESM.pdf]

Annual  
change in  
Precipitation  
(mm)

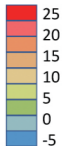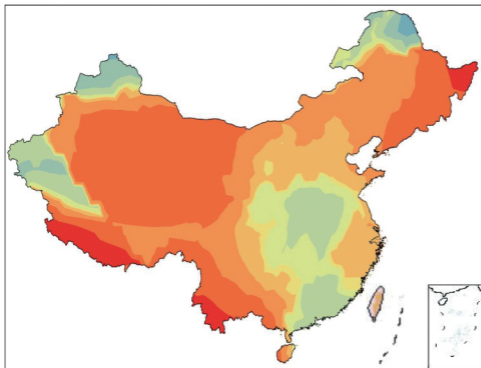

Supplement: Supplementary file 5 — Additional file 5: Figure S5. Universal Kriging estimated annual changes in precipitation (mm) in China. [file 40249_2023_1083_MOESM5_ESM.pdf]
