# Supplementary material for: Climate change and Aedes albopictus risks in China: current impact and future projection
Source: Infect Dis Poverty. 2023 Mar 24;12:26. doi: 10.1186/s40249-023-01083-2 (PMC10037799; doi:10.1186/s40249-023-01083-2)
Supplement: Supplementary file 2 — Additional file 2: Figure S2. Univariate analyses of relationship between Ae. albopictus presence and climatic variables. a) Monthly mean temperature (°C); b) Monthly maximum temperature (°C); and c) Monthly minimum temperature (°C). Acc stands for accuracy. Yale represents Yale’s association. Dash line represents the optimal cutoff of temperature. [file 40249_2023_1083_MOESM2_ESM.pdf]

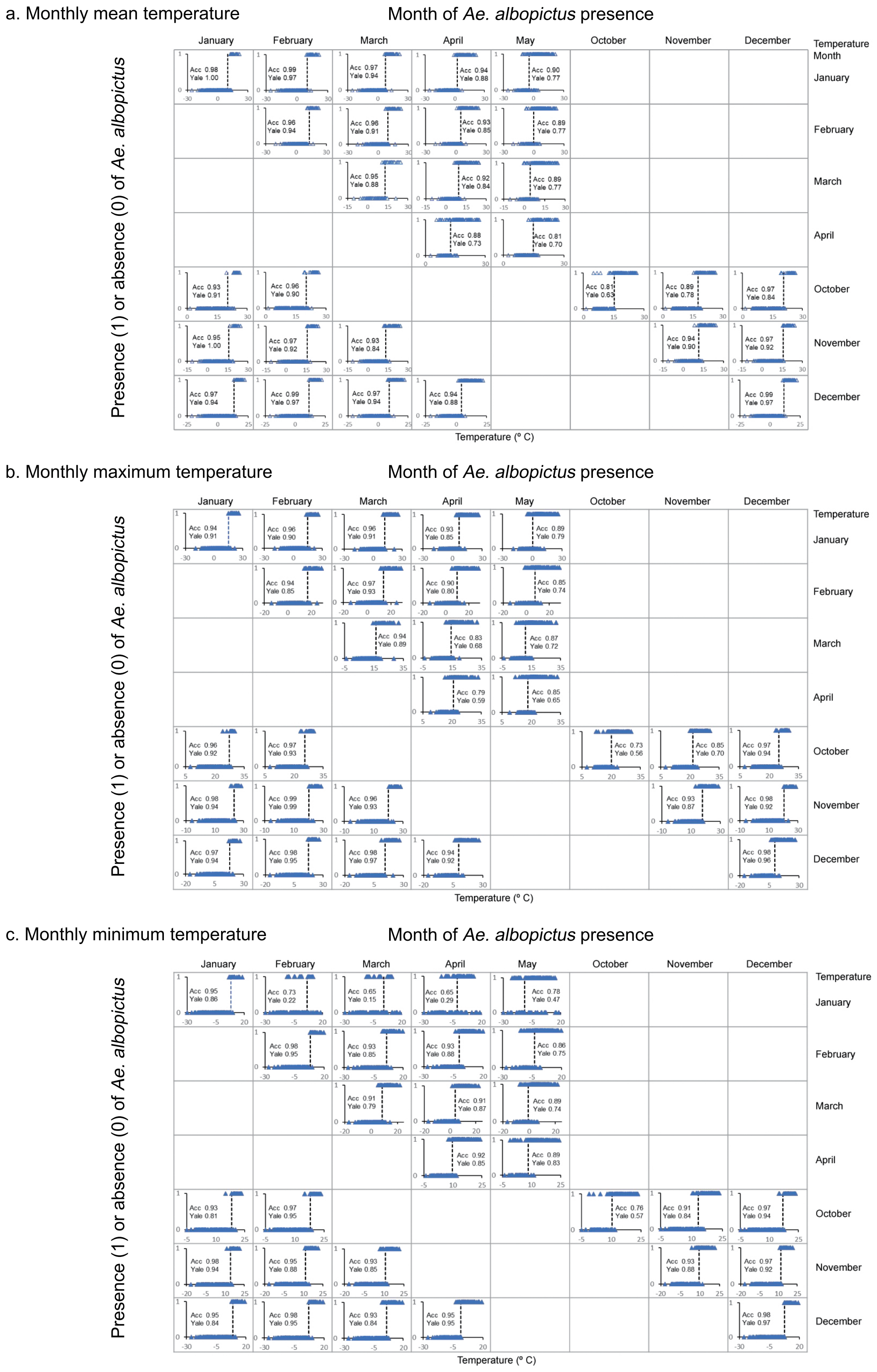
a. Monthly mean temperature
Month of Ae. albopictus presence
January
February
March
April
May
October
November
December
Temperature Month
Presence (1) or absence (0) of Ae. albopictus
Acc 0.98 Yale 1.00
Acc 0.99 Yale 0.97
Acc 0.97 Yale 0.94
Acc 0.94 Yale 0.88
Acc 0.90 Yale 0.77
Acc 0.96 Yale 0.94
Acc 0.96 Yale 0.91
Acc 0.93 Yale 0.85
Acc 0.89 Yale 0.77
Acc 0.95 Yale 0.88
Acc 0.92 Yale 0.84
Acc 0.89 Yale 0.77
Acc 0.88 Yale 0.73
Acc 0.81 Yale 0.70
Acc 0.93 Yale 0.91
Acc 0.96 Yale 0.90
Acc 0.81 Yale 0.63
Acc 0.89 Yale 0.78
Acc 0.97 Yale 0.84
Acc 0.95 Yale 1.00
Acc 0.97 Yale 0.92
Acc 0.93 Yale 0.84
Acc 0.94 Yale 0.90
Acc 0.97 Yale 0.92
Acc 0.97 Yale 0.94
Acc 0.99 Yale 0.97
Acc 0.97 Yale 0.94
Acc 0.94 Yale 0.88
Acc 0.99 Yale 0.97
Temperature (º C)
b. Monthly maximum temperature
Month of Ae. albopictus presence
January
February
March
April
May
October
November
December
Temperature
Presence (1) or absence (0) of Ae. albopictus
Acc 0.94 Yale 0.91
Acc 0.96 Yale 0.90
Acc 0.96 Yale 0.91
Acc 0.93 Yale 0.85
Acc 0.89 Yale 0.79
Acc 0.94 Yale 0.85
Acc 0.97 Yale 0.93
Acc 0.90 Yale 0.80
Acc 0.85 Yale 0.74
Acc 0.94 Yale 0.89
Acc 0.83 Yale 0.68
Acc 0.87 Yale 0.72
Acc 0.79 Yale 0.59
Acc 0.85 Yale 0.65
Acc 0.96 Yale 0.92
Acc 0.97 Yale 0.93
Acc 0.73 Yale 0.56
Acc 0.85 Yale 0.70
Acc 0.97 Yale 0.94
Acc 0.98 Yale 0.94
Acc 0.99 Yale 0.99
Acc 0.96 Yale 0.93
Acc 0.93 Yale 0.87
Acc 0.98 Yale 0.92
Acc 0.97 Yale 0.94
Acc 0.98 Yale 0.95
Acc 0.98 Yale 0.97
Acc 0.94 Yale 0.92
Acc 0.98 Yale 0.96
Temperature (º C)
c. Monthly minimum temperature
Month of Ae. albopictus presence
January
February
March
April
May
October
November
December
Temperature
Presence (1) or absence (0) of Ae. albopictus
Acc 0.95 Yale 0.86
Acc 0.73 Yale 0.22
Acc 0.65 Yale 0.15
Acc 0.65 Yale 0.29
Acc 0.78 Yale 0.47
Acc 0.98 Yale 0.95
Acc 0.93 Yale 0.85
Acc 0.93 Yale 0.88
Acc 0.86 Yale 0.75
Acc 0.91 Yale 0.79
Acc 0.91 Yale 0.87
Acc 0.89 Yale 0.74
Acc 0.92 Yale 0.85
Acc 0.89 Yale 0.83
Acc 0.93 Yale 0.81
Acc 0.97 Yale 0.95
Acc 0.76 Yale 0.57
Acc 0.91 Yale 0.84
Acc 0.97 Yale 0.94
Acc 0.98 Yale 0.94
Acc 0.95 Yale 0.88
Acc 0.93 Yale 0.85
Acc 0.93 Yale 0.88
Acc 0.97 Yale 0.92
Acc 0.95 Yale 0.84
Acc 0.98 Yale 0.95
Acc 0.93 Yale 0.84
Acc 0.95 Yale 0.95
Acc 0.98 Yale 0.97
Temperature (º C)
