# Supplementary material for: Climate change and Aedes albopictus risks in China: current impact and future projection
Source: Infect Dis Poverty. 2023 Mar 24;12:26. doi: 10.1186/s40249-023-01083-2 (PMC10037799; doi:10.1186/s40249-023-01083-2)
Supplement: Supplementary file 6 — Additional file 6: A Machine Learning Classification and Regression Trees (CART) for Aedes albopictus distribution modeling. B. Universal Kriging for spatial interpolation of annual climate changes. [file 40249_2023_1083_MOESM6_ESM.docx]

**Additional file 6**

1. **Machine Learning Classification and Regression Trees (CART) for *Aedes albopictus* distribution modeling**

Decision Trees are an important type of algorithm for predictive modeling machine learning. The CART algorithm provides a foundation for important algorithms like bagged decision trees, random forest and boosted decision trees. The CART algorithm is a type of classification algorithm that is required to build a decision tree based on Gini’s impurity index.

**Greedy Splitting**

Creating a CART model involves selecting input variables and splitting data points on those variables until a suitable tree is constructed. The selection of which input variable to use and the specific split or cutoff-point is chosen using a greedy algorithm to minimize a cost function.

For classification trees, the Gini index function is used which provides an indication of how the training data assigned to each node (tree branch):

$$G=\sum_{k} (p_{k}(1-p_{k}))$$

where G is the Gini index over all classes, $p_{k}$ are the proportion of training samples with class *k* in the space of interest. A node that has all classes of the same type (perfect class purity) will have G = 0; while G = 0.5 which is the maximum if there is a 50-50 split of classes for a binary classification.

In our study, we used presence (1) and absence (0) of *Ae. albopictus*, therefore it’s a binary classification, i.e., G is

$$G=1-p_{0}^{2}-p_{1}^{2}$$

Assuming Y is the presence/absence observations of *Ae. albopitus*, X = (x_1_, x_2_, …, x_n_) is the dataset of independent variables. Here are how the Y records are split recursively. At each node (parent class) *t*, some records are split to the right ($t_{R}$) and others to the left ($t_{L}$) branches based on the search of all independent variables in sample space X. Assuming $P_{R}$ and $P_{L}$ are the proportions of the records classified to the right and left, respectively, the candidate independent variable $x_{i}$ for the next split (*s*) is searched all over the X space, the one minimize the following cost function will be the variable for the next split, i.e., minimize

$$G\left( s | t \right)=2P_{L}P_{R}\sum_{j}^{k} |P\left( j | t_{L} \right)-P(j|t_{R)}|$$

where *k* is the class count, $P(j|t_{L})$ and $P(j|t_{R})$ are the proportions of class j records in $t_{L}$ and $t_{R}$.

The process starts with the whole dataset at *t* = 0. For each variable, an optimal cutoff is selected to minimize the *G* value, the variable that gives the least *G* value is selected and sample dataset is split based on the cutoff of the variable. The process continues with the two split datasets from *t* = 0, until the stopping rules are reached.

**Stopping and Pruning Criterion**

To avoid overfitting of training data and poor performance of test data, a stop rule is required. The most common stopping procedure is to use a minimum count on the number of training samples at each node. In this study we used 20 and 5 records rules, i.e., if a node has < 20 samples, it will not be further split; and a tree leaf node will be removed if it contains < 5 samples or no further improvements in cost function can be made.

A CART model was built separately for each month.

**Reference**

Breiman L, Friedman JH, Olshen RA, Stone CJ. (1984). Classification and regression trees. Monterey, CA: Wadsworth & Brooks/Cole Advanced Books & Software.

1. **Universal Kriging for spatial interpolation of annual climate changes**

To obtain the distribution of changes in annual rates of minimum/maximum/mean temperature and annual precipitation across China, we used Universal Kriging (UK) algorithms to interpolate the data from the 90 meteorological stations. Based on previous study of temperature changes in Arctic, we assumed the temperature increase depending on the latitude, thus a regression-Kriging is required. UK is one of the Kriging methods which allows for the modeling of spatial trends (external) in addition to spatial interpolations.

Let Z(u) be an annual rate of change of a climatic variable, the expected (mean) value is

$$m\left( u \right)=E[Z(u)]$$

where *u* is the longitude/latitude of a given point and *E*(·) denotes expected value. Assuming a polynomial drift model of $f_{i}(u)$, then

$m\left( u \right)=\sum_{i=0}^{n} a_{i}f_{i}(u)$, with $f_{0}\left( u \right)=1$

We used the most common form of $f_{i}(u)$, i.e., maximum of 3-orders of polynomial of latitude (*y*) and longitude (*x*),

$$m\left( u \right)=a_{0}+a_{1}x+a_{2}y+a_{3}x^{2}+a_{4}xy+\ldots+a_{8}xy^{2}+a_{9}y^{3}$$

Assuming Y(u) are the observations (estimated annual rate of change based on observed trends), at any given location $u_{0}$, an unbiased estimator $\hat{Z}(u_{0})$ for $Z(u_{0})$ is defines as

$\hat{Z}\left( u_{0} \right)-Z\left( u_{0} \right)=\sum_{i=1}^{k} a_{i}Y\left( u_{i} \right)-Y(u_{0})$

The weights $a_{i}$ can be estimated by minimizing the following Lagrange function

$L\left( a_{i},\mu_{j} \right)=\sigma^{2}\left( u_{0} \right)+2\mu_{0}\left( \sum_{i=1}^{k} a_{i}-1 \right)+2\sum_{j=1}^{n} \mu_{j}\left( \sum_{i=1}^{k} a_{i}f_{i}\left( u_{i} \right)-f_{j}(u_{0}) \right)$

where $\sigma^{2}\left( u_{0} \right)=\sum_{i=1}^{k} \sum_{j=1}^{k} a_{i}a_{j}Cov(Y\left( u_{i} \right)-Y\left( u_{0} \right),Y\left( u_{j} \right)-Y(u_{0}))$ , *Cov* is the co-variance between the two locations.

1. The weights that produce the minimum estimation variance are the solution to the following equation system

$\sum_{i=1}^{k} a_{i}\gamma\left( u_{i},u_{1} \right)-\mu_{0}-\sum_{j=1}^{n} \mu_{j}f_{j}\left( u_{1} \right)=\gamma(u_{0},u_{1})$

$\sum_{i=1}^{k} a_{i}\gamma\left( u_{i},u_{2} \right)-\mu_{0}-\sum_{j=1}^{n} \mu_{j}f_{j}\left( u_{2} \right)=\gamma(u_{0},u_{2})$

……

$\sum_{i=1}^{k} a_{i}\gamma\left( u_{i},u_{k} \right)-\mu_{0}-\sum_{j=1}^{n} \mu_{j}f_{j}\left( u_{k} \right)=\gamma(u_{0},u_{k})$

$\sum_{i=1}^{k} a_{i}=1$

$\sum_{i=1}^{k} a_{i}f_{1}\left( u_{i} \right)=f_{1}(u_{0})$

$\sum_{i=1}^{k} a_{i}f_{2}\left( u_{i} \right)=f_{2}(u_{0})$

……

$\sum_{i=1}^{k} a_{i}f_{k}\left( u_{i} \right)=f_{k}(u_{0})$

Where $\gamma\left( u_{i},u_{j} \right)=Cov(Y\left( u_{i} \right)-Y\left( u_{0} \right),Y\left( u_{j} \right)-Y(u_{0}))$.

1. The minimum squared error of the estimation is

$\sigma^{2}\left( u_{0} \right)=\sum_{i=1}^{k} a_{i}\gamma\left( u_{i},u_{j} \right)-\mu_{0}-\sum_{j=1}^{n} \mu_{j}f_{j}(u_{0})$

**References**

Rantanen M, Karpechko AY, Lipponen A, Nordling K, Hyvärinen O, Ruosteenoja K, et al. The Arctic has warmed nearly four times faster than the globe since 1979. Commun Earth Environ. 2022;3:168.

Webster R, Oliver MA. Geostatistics for environmental scientists. 2^nd^ ed. Chichester: Wiley; 2007.

Pebesma EJ. The role of external variables and GIS databases in geostatistical analysis. Trans GIS. 2006;10(4):615–632.
